# The receptor-like kinase SOBIR1 interacts with *Brassica napus* LepR3 and is required for *Leptosphaeria maculans* AvrLm1-triggered immunity

**DOI:** 10.3389/fpls.2015.00933

**Published:** 2015-10-29

**Authors:** Lisong Ma, M. Hossein Borhan

**Affiliations:** Saskatoon Research Centre, Agriculture and Agri-Food Canada, SaskatoonSK, Canada

**Keywords:** receptor-like protein, LepR3, SOBIR1, *BAK1/SERK3*, *Brassica napus*, *Leptosphaeria maculans*

## Abstract

The fungus *Leptosphaeria maculans* (*L. maculans*) is the causal agent of blackleg disease of canola/oilseed rape (*Brassica napus*) worldwide. We previously reported cloning of the *B. napus* blackleg resistance gene, *LepR3*, which encodes a receptor-like protein. LepR3 triggers localized cell death upon recognition of its cognate Avr protein, AvrLm1. Here, we exploited the *Nicotiana benthamiana* model plant to investigate the recognition mechanism of AvrLm1 by LepR3. Co-expression of the *LepR3/AvrLm1* gene pair in *N. benthamiana* resulted in development of a hypersensitive response (HR). However, a truncated *AvrLm1* lacking its indigenous signal peptide was compromised in its ability to induce *LepR3*-mediated HR, indicating that AvrLm1 is perceived by LepR3 extracellularly. Structure-function analysis of the AvrLm1 protein revealed that the C-terminal region of AvrLm1 was required for *LepR3*-mediated HR in *N. benthamiana* and for resistance to *L. maculans* in *B. napus*. LepR3 was shown to be physically interacting with the *B. napus* receptor like kinase, SOBIR1 (BnSOBIR1). Silencing of *NbSOBIR1* or *NbSERK3 (BAK1)* compromised LepR3-AvrLm1-dependent HR in *N. benthamiana*, suggesting that *LepR3*-mediated resistance to *L. maculans* in *B. napus* requires *SOBIR1* and *BAK1/SERK3*. Using this model system, we determined that BnSOBIR1 and SERK3/BAK1 are essential partners in the LepR3 signaling complex and were able to define the AvrLm1 effector domain.

## Introduction

Plants mainly rely on induced innate immune system to resist pathogen infection. Membrane-localized pattern-recognition receptors (PRRs) form the first layer of defense by detecting conserved microbe or pathogen-associated molecular patterns (MAMPs or PAMPs) and activating PAMP-triggered immunity (PTI; Jones and Dangl, 2006). One of the PRRs that has been studied extensively is FLAGELLIN SESING 2 (FLS2) that recognizes the bacterial PAMP flagellin 22 (flg22; Cook et al., 2015). FLS2 is a leucine-rich repeat receptor-like kinase (LRR-RLK) and requires another LRR-RLK, the brassinosteroid receptor BRASSINOSTEROID INSENSITIVE 1 -ASSOCIATED KINASE 1 (BAK1)/SERK3, for perception of flg22 (Ben Khaled et al., 2015). Specialized pathogens have evolved strategies to overcome PTI by secretion of effector proteins. Most effector proteins are translocated into the plant cell where they modulate basal immunity and promote pathogen infection (Feng and Zhou, 2012). To counteract their activity, plants have evolved resistance (R) proteins to detect pathogen effectors, which leads to the activation of effector-triggered immunity (ETI; Jones and Dangl, 2006; Thomma et al., 2011). The majority of R proteins are cytoplasmic, but there are several examples of receptor-like proteins (RLP) with trans-membrane and extracellular LRR domains. The most widely studied examples of RLPs are the tomato Cf proteins (e.g., Cf2, Cf4, Cf9) that recognize corresponding effector proteins (e.g., Avr2, Avr4, Avr9) secreted by the fungus *Cladosporium fulvum*, the cause of tomato leaf mold disease (Rivas and Thomas, 2005). Accumulating evidence suggests that overlap exists between the PRR perception of PAMPs and the RLP recognition of effectors secreted by the apoplastic fungi. A recent report by Postma et al. (2015) showed that upon induction by Avr4, Cf4, interacts with BAK1. The recently discovered transmembrane LRR-RLK, SUPPRESSOR OF BIR1-1 (SOBIR1), is also required for ETI initiated by RLPs (Liebrand et al., 2013). SOBIR1, which was initially identified as a suppressor of BIR1 (BAK1-interacting receptor-like kinase 1), is conserved throughout the plant kingdom (Gao et al., 2009). SOBIR1 is required for Cf2-, Cf4-, and Ve1-mediated HR in tobacco and resistance of tomato against the fungal pathogens *C. fulvum* and *Verticillium dahlia*, respectively (Gao et al., 2009; Liebrand et al., 2013).

*Leptosphaeria maculans* (*L. maculans*) is the causative agent for blackleg disease (phoma stem canker) on *Brassica* species and is a major pathogen of *Brassica napus* (oilseed rape, canola) crops worldwide (West et al., 2001). *L. maculans* ascospores are released from infected canola stubble and infect the leaves and cotyledons of canola seedlings (Rouxel and Balesdent, 2005). During leaf colonization, *L. maculans* remains extracellular, similar to *C. fulvum* infection on tomato. Resistance against *L. maculans* in canola seedlings stage is race-specific. To date, 16 race-specific resistance (*R*) genes against *L. maculans* have been identified from *Brassica* species (Raman et al., 2013), but only two *R* genes, *LepR3*, and *Rlm2*, have been cloned (Larkan et al., 2013, 2015). LepR3 recognizes the *L. maculans* effector AvrLm1 (Larkan et al., 2013) and the *AvrLm2* gene which encodes the effector corresponding to Rlm2 has been cloned, (Ghanbarnia et al., 2015). *LepR3* and *Rlm2* are allelic and encode membrane-bound LRR-RLPs. We recently reported that *B. napus* Rlm2 interacts with the *Arabidopsis thaliana* SOBIR1 (Larkan et al., 2015), suggesting SOBIR1 is a component of LRR-RLP-mediated resistance against *L. maculans*.

Recognition of an AVR protein by its cognate R protein often culminates into a hypersensitive response (HR) around the infection site, a phenotype that can also be produced by co-expressing both genes in the model plant tobacco using agroinfiltration (Goodin et al., 2008; Ma et al., 2012). Such transient assays in tobacco have been used to analyze the function of *R/AVR* gene pairs. For example, co-expression of the gene pairs *Cf2/Avr2, Cf4/Avr4*, and *Cf9/Avr9* from tomato-*C. fulvum* and *Ve1/Ave1* from tomato-*V. dahlia* plant pathogen systems trigger HR in *Nicotiana benthamiana* leaves (van der Hoorn et al., 2000; Cai et al., 2001; Zhang et al., 2012). To overcome the limitation of *B. napus* for functional analysis of R/Avr proteins, we exploited the *N. benthamiana* model system to investigate newly discovered *R/AVR* gene pairs from the *B. napus-L. maculans* pathosystem. Here we use this system, to identify the AvrLm1effector domain. We provide evidence that *B. napus* SOBIR1 interacts with LepR3 and is required for *LepR3-*mediated cell death, indicating that BnSOBIR1 forms a signaling complex with LepR3 in *B. napus* to initiate the innate immunity response upon recognition of AvrLm1. Finally, we determined that LepR3-AvrLm1-mediated cell death in *N. benthamiana* is also dependent on SERK3/BAK1.

## Materials and Methods

### Plant Materials and *L. maculans* Isolate

The susceptible doubled-haploid (DH) line Topas DH16516 and the *LepR3* transgenic line NLA8 that were used in this study have been described elsewhere (Larkan et al., 2013). *L. maculans* isolate 3R11 was used for pathology tests and transformation. The genotype of the single-spore *L. maculans* isolate 3R11 has been described by Ghanbarnia et al. (2015).

### Generation of Transgenic *L. maculans* Strains

To generate a *L. maculans* Gateway^TM^ compatible vector in which the designated gene was driven by *AvrLm1* native promoter, the promoter region of *AvrLm1* was amplified by PCR with the AvrLm1-Up-F and AvrLm1-Up-R primers (see Supplementary Table S1). The resulting amplicon was cloned into the *Acc*65I site of the GW-pPk2 vector (Larkan et al., 2013). The *TubA* terminator was amplified with the primer pair TubA-F/TubA-R and cloned into the *Pac*I site of GW-pPk2. The resulting vector was named GW-pLM4. AvrLM1 truncated variants, AvrLm1 Δ40, Δ50, and CTΔ14, with Gateway^TM^ *attB* linkers were synthesized by GenScript (GeneScript, USA). The synthesized genes were introduced into the entry vector pDONR/Zeo then into the binary vector GW-pLM4 following the Gateway^TM^ protocol (Life Technologies, USA). The plasmids were transformed into *Agrobacterium tumefaciens* AGL1 and used for subsequent *Agrobacterium*-mediated transformation of pycnidiospores from the *L*. *maculans* isolate 3R11 (*avrLm1*) as described by Utermark and Karlovsky (2008). Supplementary Table S1 contains the sequences for all the primers used in this study.

### Binary Vector Constructions

For transient expression, the *LepR3* ORF was amplified using the primer pair LepR3-FB and LepR3-RB-S/+S. Full length *AvrLm1* was amplified by PCR- from *L. maculans*-*B. napus* cDNA using primers AvrLm1-FB and AvrLm1-RB and RNA isolated from the young cotyledons of *B. napus* cv. Topas infected with *L. maculans* v23.1.3. Truncated *AvrLm1* lacking the region encoding the signal peptide was amplified using primers ΔspAvrLm1-FB and AvrLm1-RB. The *PR1a-AvrLm1* gene with Gateway^TM^*attB* linkers was synthesized by GenScript (GeneScript, USA). DNA fragments were introduced into the Gateway^TM^ entry vector pDONR/Zeo (Life Technologies, USA) to produce pENTR/Zeo:*LepR3*, pENTR/Zeo:*LepR3+S* (with stop codon after the coding region), pENTR/Zeo:*AvrLm1* and pENTR/Zeo:Δsp*AvrLm1* constructs which were then transferred to the binary vector pEarleyGate100 (Earley et al., 2006). The resulting plasmids were transferred into the *A. tumefaciens* strain GV3101 (pMP90).

For co-immunoprecipitation (co-IP) studies, the pENTR/Zeo:*LepR3* was recombined into the binary vector pGWB414 containing the C-terminal Human influenza hemagglutinin (HA) tag (Nakagawa et al., 2007) resulting in pGWB414:*LepR3-HA*. Full length *BnSOBIR1-A3* and *BnSOBIR1-C3* were amplified by PCR- from the *L. maculans*-*B. napus* cDNA using the primers BnSOBIR1A3C3-FB and BnSOBIR1-A3C3-RB-S. The amplified fragments were introduced into the Gateway^TM^entry vector pDONR/Zeo (Life Technologies, USA) resulting in the constructs pENTR/Zeo:*BnSOBIR1-A3* and pENTR/Zeo:*BnSOBIR1-C3*. These were recombined into the binary vector pGWB417 which contains a C-terminal Myc tag. The binary plasmids were transferred into the *A. tumefaciens* strain GV3101 (pMP90).

### *Agrobacterium*-mediated Transient Assay in *N. benthamiana*

*Agrobacterium*-mediated transient expression in *N. benthamiana* was performed according to the method described previously (Ma et al., 2013). Briefly, *Agrobacterium* was grown to an absorbance of 0.8 at OD_600_ in LB-mannitol medium supplemented with 20 μM acetosyringone and 10 mM MES (pH 5.6). Cells were pelleted by centrifugation at 3500 *g* for 20 min and then re-suspended in infiltration medium (1x MES, 10 mM MES pH 5.6, 2% w/v sucrose, 200 μM acetosyringone). The gene encoding the silencing suppressor, p19 from the Tomato bushy stunt virus (Voinnet et al., 2003), was co-expressed in all the transient expression assays. Leaves of 4–5 weeks old *N. benthamiana* plants were infiltrated with *Agrobacterium* culture at OD_600_ of 2.0 and the triple-infiltration was done with a 1:1:1 mix ratio of *Agrobacterium*. Total 30 leaves of *N. benthamiana* were infiltrated and occurrence of HR was calculated on the sites that *PR1a-AvrLm1/LepR3* was co-infiltrated. Representative leaves showing HR were photographed 3 or 6 days after infiltration.

### Co-immunoprecipitation and Western Blotting

Co-IP was performed as described by Liebrand et al. (2013). Briefly, total proteins were extracted from *N. benthamiana* leaves 48 h after infiltrating with a mixture of *A. tumefaciens* GV3101 containing either pGWB414:*LepR3* or pGWB417:*BnSOBIR1-A3* or pGWB417:*BnSOBIR1-C3* or *p19* in buffer [150 mM NaCl, 1.0% IGEPAL CA-630 (NP-40), 0.5% sodium deoxycholate, 0.1% SDS, 50 mM Tris (pH 8.0) 1x complete protease inhibitor cocktail (Roche, USA)]. Extracts were centrifuged at 18,000 *g*, 4°C for 15 min, and 2 ml of supernatant was collected and applied to 50 μl of Anti-HA magnetic beads (Pierce, USA), which was then incubated for 2 h at 4°C in a rotator. After washing the beads four times with extraction buffer, immuno-precipitated proteins were separated on an 8% SDS-PAGE gel and transferred to overnight to a PVDF membrane using wet blotting (Bio-Rad, USA). Skimmed milk powder (5%) was used as a blocking agent. A 1:2000 dilution of anti-HA antibody (Pierce, USA) or 1:2000 diluted anti-Myc antibody (cMyc 9E10, sc-40-HRP, Santa Cruz) was used. The goat-anti-mouse secondary antibody (Pierce, USA) was used as a 1:15000 dilution. The luminescent signal was visualized using Immobilon Western Chemiluminescent HRP Substrate and BioMax MR film (Kodak).

### TRV-mediated Gene Silencing in *N. benthamiana* Plants and Hypersensitive Response Assay

Virus-induced gene silencing (VIGS) was performed using the tobacco rattle virus (TRV)-mediated gene silencing vector pTRV1 (Ratcliff et al., 2001) and pTRV2 constructs: *pTRV2:GFP* (Burch-Smith et al., 2006), *pTRV2:PDS* (Liu et al., 2002), *pTRV2:NbSBOBIR1* (Liebrand et al., 2013), and *pTRV2:NbSERK3a/b* (Chaparro-Garcia et al., 2011). The VIGS experiments in *N. benthamiana* were performed as described previously (Gabriels et al., 2006). In brief, cotyledons of 2-week-old *N. benthamiana* seedlings were infiltrated with the pTRV1 and pTRV2 constructs in a 1:1 ratio. Agroinfiltration was performed as described by Ma et al. (2012). For monitoring the development of HR, 3 weeks after TRV infiltration, mature leaves were co-infiltrated to express *PR1a-AvrLm1*/*LepR3, PR1a-AvrLm1*/*GFP, LepR3*/*GFP*, Bcl2-Associated proteinX(BAX; Lacomme and Santa Cruz, 1999)/*GFP*, respectively. All the co-infiltrations were performed in a 1:1 mix ratio of *Agrobacterium* containing the corresponding construct at OD_600_ = 1. Total 30 leaves of *N. benthamiana* were infiltrated and occurrence of HR was calculated on the sites that *PR1a-AvrLm1/LepR3* was co-infiltrated. Six days after infiltration, leaves were examined for development of an HR and representative leaves were photographed. The experiment was repeated three times.

### Quantitative RT-PCR Analysis

For qRT-PCR, total RNA was isolated from *N. benthamiana* at 2 weeks after agro-inoculation with the various VIGS constructs including TRV:*GFP* and TRV:*NbSOBIR1*. Wildtype (WT) *N. benthamiana* leaves without TRV inoculation was collected for the negative control as well. The *N. benthamiana* leaves were ground in liquid nitrogen. Total RNA from the samples was extracted with TRIzol LS reagent (Invitrogen, USA) and subsequently purified with RNAeasy Mini kit (Qiagen, USA). DNA was removed by on-column treatment with RNase-free DNase (Qiagen, USA). cDNA was synthesized using SuperScript III first-strand synthesis SuperMix kit according to the manufacture’s protocol (Invitrogen, USA). qRT-PCR was performed using a 7700 real-time PCR machine (Applied Biosystems) and SsoFast EvaGreen Supermix (BIO-RAD). RT-PCR was performed for the *NbSBOIR1 gene* with three biological samples. Expression of *NbSOBIR1* was investigated using primer NbSOBIR1-to266 and NbSOBIR1-to267 (Liebrand et al., 2013). Expression of endogenous actin was used to calibrate the expression level of the query genes, as previously described (Liebrand et al., 2013). The primers used for qRT-PCR are described in Supplementary Table S1. *Ct* values were analyzed according to the 2^-ΔΔCt^ method (Livak and Schmittgen, 2001). The statistical significance of differences was calculated using GraphPad Prism 6 (GraphPad Software, Inc., USA) with One-way ANOVA followed by the Turkey post-test to obtain the *P*-value. Data are shown as mean ± SEM of three biological replicates from one representative experiment. Significant differences between treatments and controls are represented by three asterisks (*P* < 0.001).

## Results

### AvrLm1 Triggers LepR3-mediated Cell Death in *N. benthamiana*

**FIGURE 1:**
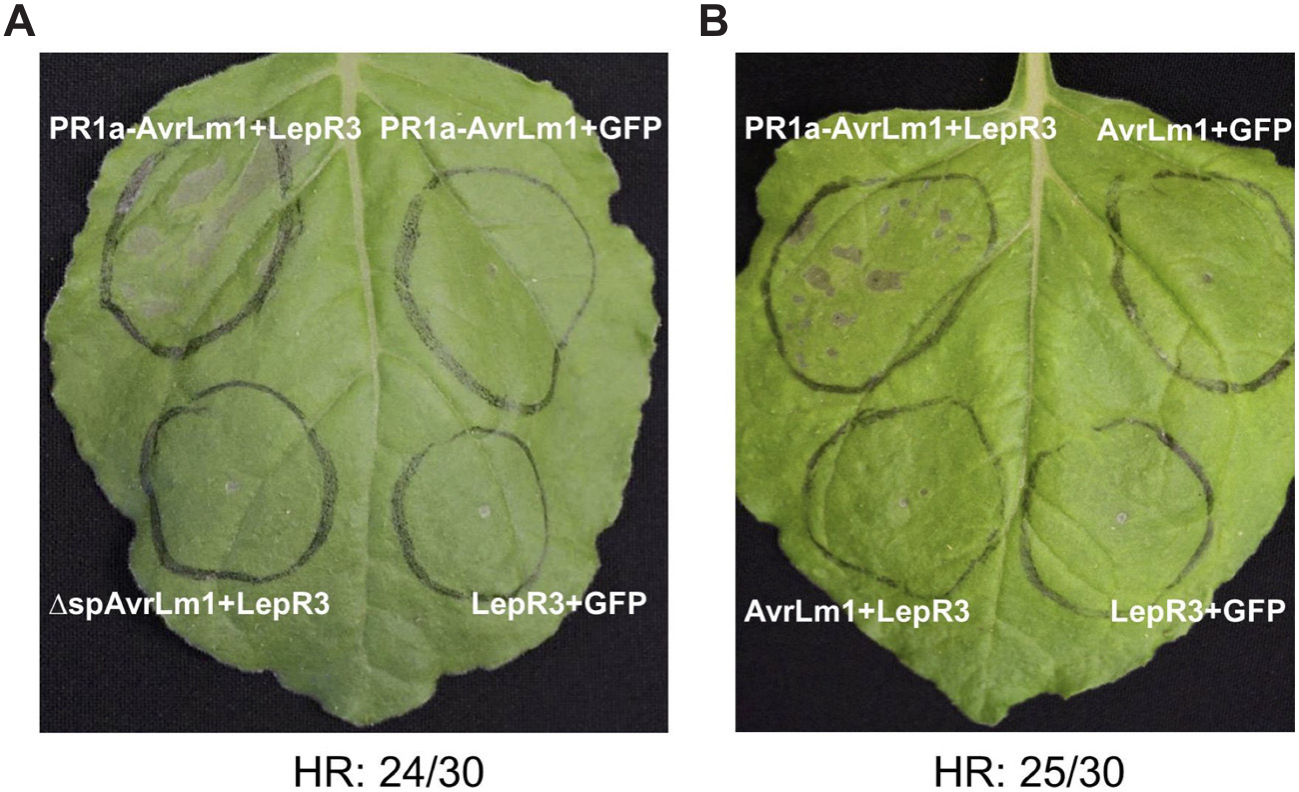
**Transient expression of *AvrLm1* triggers *LepR3*-mediated cell death in *Nicotiana benthamiana* leaves. (A)** *N. benthamiana* leaves Agro-infiltrated with the *LepR3, PR1a*-*AvrLm1*, and *GFP* constructs in the order indicated on the image. To ensure secretion of AvrLm1 to the apoplastic space, the indigenous AvrLm1 signal peptide was replaced by the tobacco PR1a signal peptide and the construct was designated as PR1a-AvrLm1. Truncated AvrLm1 lacking its indigenous signal peptide (ΔspAvrLm1) was also included. Co-expression of *LepR3* or *PR1a*-*AvrLm1* with *GFP* served as negative controls. The experiment was performed three times and each time 10 plants were infiltrated. **(B)** *N. benthamiana* leaves Agro-infiltrated with *PR1a-AvrLm1/LepR3, AvrLm1/LepR3, AvrLm1/GFP, and LepR3/GFP* constructs in the order indicated in the image. The numbers under the panels indicate the total occurrence of HR within 30 sites co-infiltrated with *PR1a-AvrLm1/LepR3*. Images are from photographs taken 6 days after infiltration.

Transient expression of *R* genes and their cognate effectors (pathogen *Avr* genes) in *N. benthamiana* often leads to a HR and is a commonly used tool for the functional analysis of *R-Avr* genes (van der Hoorn et al., 2000; Ma et al., 2012). To determine if perception of AvrLm1 by LepR3 triggers HR in *N. benthamiana, AvrLm1*, and *LepR3* were ectopically co-expressed in leaves using Agro-infiltration. *L. maculans* is confined to the apoplastic space, therefore to ensure efficient secretion of AvrLm1 into the apoplast the native signal peptide of AvrLm1 was replaced with the tobacco PR1a signal peptide (van Esse et al., 2006); this construct was designated *PR1a-AvrLm1*. The PR1a signal peptide has been widely used for secretion of plant pathogen effectors into the extracellular space (van Esse et al., 2007). The wild type AvrLm1 containing its indigenous signal peptide and the Δsp*AvrLm1* construct encoding a truncated AvrLm1 protein without its signal peptide were included in this assay as well. *A. tumefaciens* harboring either *PR1a-AvrLm1*, wild type *AvrLm1* or Δsp*AvrLm1* was co-infiltrated with an *A. tumefaciens* strain carrying *B. napus LepR3*. Co-infiltration of either *AvrLm1* or *LepR3* with *GFP* served as the negative control. 80% of the sites infiltrated with *PR1a-AvrLm1* showed a *LepR3*-dependent HR approximately 6 days after co-infiltration (**Figure 1A**). The wild type *AvrLm1* and Δsp*AvrLm1* did not trigger HR when co-expressed with *LepR3* (**Figures 1A,B**). Expression of *AvrLm1* or *LepR3* alone did not cause HR. The lack of HR by the full length AvrLm1 (native signal peptide) is likely due to inefficient secretion of AvrLm1 as directed by its native signal peptide in *N. benthamiana* plants. These results indicate that AvrLm1 was able to activate LepR3 leading to the development of HR and that the recognition occurred outside the plant cell which is in accordance with LepR3 being a cell surface receptor.

### The N-terminal Region of AvrLm1 is Dispensable for *LepR3*-mediated Cell Death and Resistance

**FIGURE 2:**
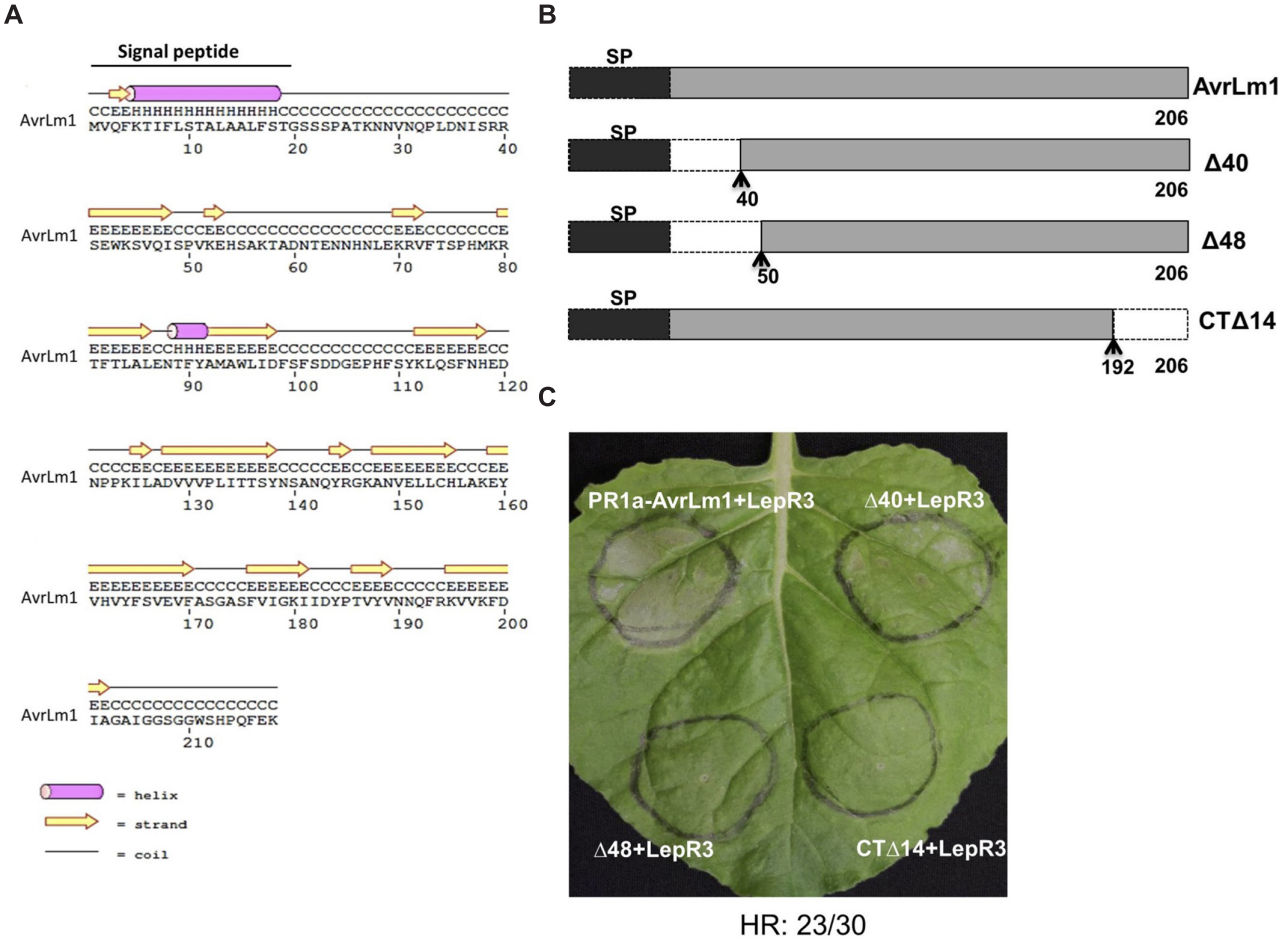
**A small N-terminal region of AvrLm1 is dispensable for *LepR3*-mediated cell death. (A)** Secondary structure prediction of the full length AvrLm1 protein. **(B)** Schematic diagram showing AvrLm1 truncations and their sizes. The signal peptide is shown as black boxes. The N- and C-terminal truncation sites are indicated by dashed lines with arrows showing the position of amino acid at the boundary between the deleted fragment and its adjacent remaining peptide segment. **(C)** *N. benthamiana* leaves were co-infiltrated with *Agrobacterium* cultures containing truncated *AvrLm1* and *LepR3* constructs. Images were taken at 6 days after infiltration. H means protein secondary structure helix, C means coli, and E means strand. The experiment was performed three times and each time with 10 plants. The numbers below the panels indicate the occurrence the occurrence of HR observed within 30 sites co-infiltrated with *PR1a-AvrLm1/LepR3* or Δ*40/LepR3*.

N-terminal and C-terminal truncated *AvrLm1* constructs were generated to define the minimal region of AvrLm1 required to trigger LepR3-mediated cell death. Design of the *AvrLm1* truncated constructs was aided by prediction of AvrLm1 secondary structure using PSIPRED (Buchan et al., 2010; **Figure 2A**). Based on the predicted secondary structure, three variants were constructed: AvrLm1-Δ40, in which the first predicted random coil after signal peptide was deleted; AvrLm1-Δ48, in which the deletion extended to the end of the predicted β-strand structure; AvrLm1-CTΔ14, which carried a deletion of the last β-strand at its C-terminus (**Figure 2B**). The tobacco PR1a signal peptide was fused to the N-terminus of the truncated constructs to allow efficient secretion of the AvrLm1 protein into the extracellular space. Each of the truncated *AvrLm1* was co-expressed with the full length *LepR3* in *N. benthamiana* leaves by Agro-infiltration. Infiltration of the *PR1a*-*AvrLm1* together with *LepR3* served as a positive control. AvrLm1-Δ40 induced a HR response similar to the full length *PR1a-AvrLm1*; however, the other truncated variants, AvrLm1-Δ48 and AvrLm1-CTΔ14, were impaired in inducing LepR3-dependent cell death (**Figure 2C**).

**FIGURE 3:**
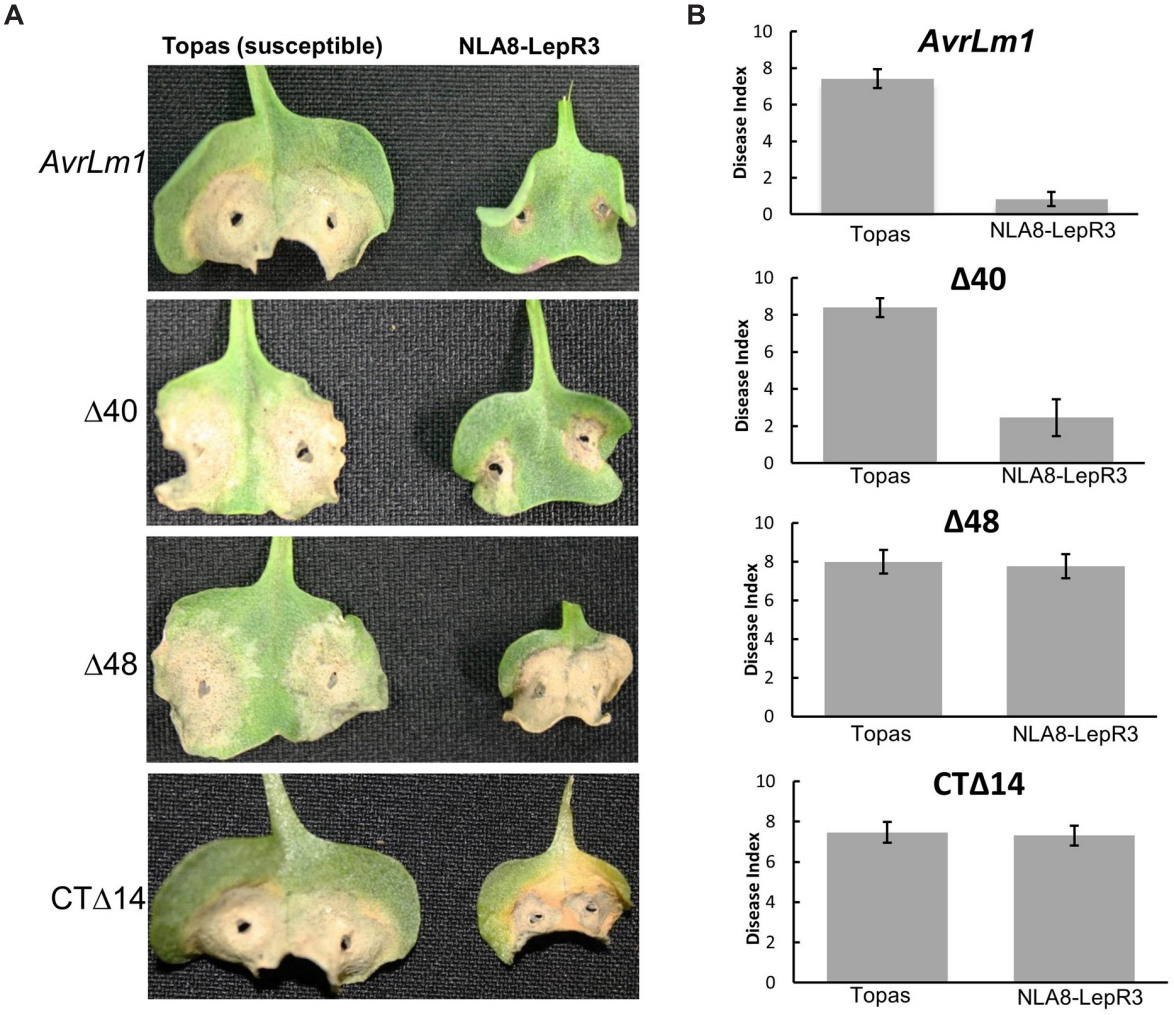
**A small N-terminal region of AvrLm1 is not required for LepR3-mediated resistance in *Brassica napus*.** Cotyledons of 7-day-old seedlings of susceptible *B. napus* Topas and *LepR3-*trasgenic NLA8 line were inoculated with a transgenic 3R11 *Leptosphaeria maculans* isolate carrying *AvrLm1* or its truncated variants (Δ40, Δ48 and CTΔ14). Lesion development (disease index from 0 to 9 with 0 showing no lesion) was scored at 14 days after inoculation. Error bars indicate standard deviation. **(A)** Images taken from infected seedlings 14 days after inoculation. **(B)** Average disease index representing data collected from 12 cotyledons of the susceptible *B. napus* cv Topas and the Topas *LepR3*-transgenic plants (NLA8).

The truncated and full length AvrLm1 with native signal peptide were transferred into the *L. maculans* isolate 3R11, which lacks the entire *AvrLm1* ORF to investigate the role of these truncated variants in *LepR3*-mediated resistance in the natural host plant *B. napus*. It should be noted that all the truncated *AvrLm1* constructs used for transformation of 3R11 contained the AvrLm1 native signal peptide and were driven by the native AvrLm1 promoter. Six transformants were selected for analysis from each transformation event. Transgenic 3R11 lines were inoculated on the cotyledons of 1-week-old seedlings of the *B. napus* cultivars Topas DH16516 (susceptible to *L. maculans*) and *LepR3-*transgenic NLA8 (a Topas DH16516–LepR3 transgenic line described by Larkan et al. (2013). Disease development was scored 14-days after inoculation. All of the *L. maculans* transgenic 3R11 were virulent and caused fully expanded lesions (similar to the wild type 3R11) on the susceptible Topas DH16516 plants (**Figure 3**) confirming that transformation of 3R11 did not affect its overall virulence. When tested on the *LepR3*-transgenic NLA8 plants, transgenic 3R11 carrying either wild type *AvrLm1* or *AvrLm1*-Δ*40* were avirulent. However, the transgenic 3R11 carrying *AvrLm1*-Δ*48* or *AvrLm1-CT*Δ*14* were virulent and caused extensive lesions similar to the response of susceptible control lines, confirming that both of these truncated AvrLm1 proteins failed to activate *LepR3*-mediated resistance. These findings confirmed the functional assay of AvrLm1 constructs in *N. benthamiana*. Taken together, these results showed that N-terminal region of AvrLm1 (or at least the first 18 amino acids of the mature AvrLm1 protein) is not required for *LepR3*-mediated resistance.

### LepR3 Interacts with *B. napus* AtSOBIR1 Homologs *in planta*

**FIGURE 4:**
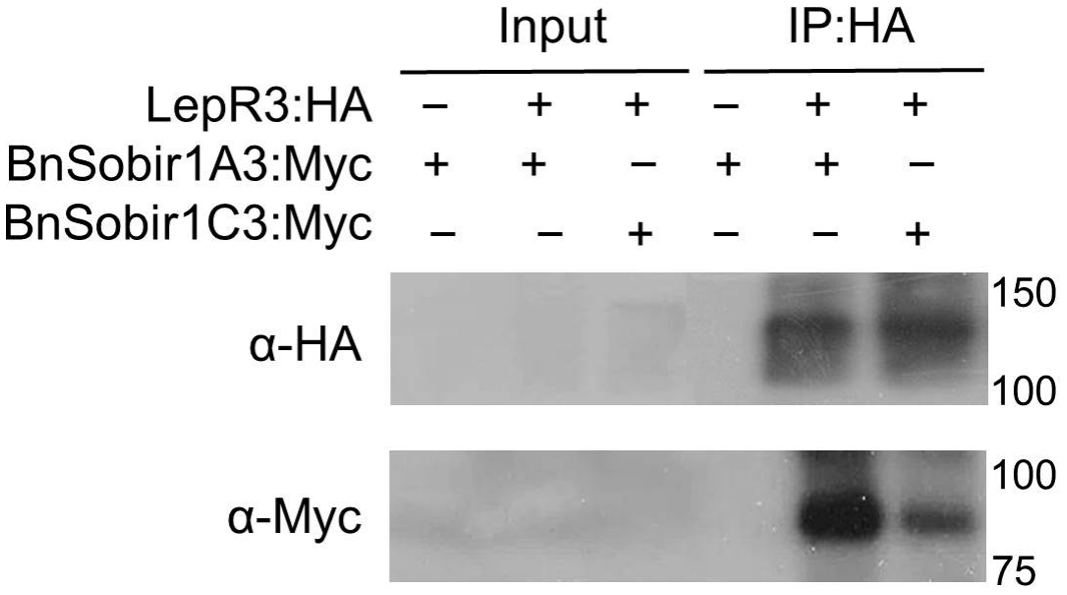
**LepR3 interacts with BnSOBIR1s *in planta*.** HA-tagged LepR3 and Myc-tagged *Bn*SOBIR1s (BnSOBIR1-A3 and BnSOBIR1-C3) were co-expressed in *N. benthamiana*. Proteins were extracted after 48 h and subjected to immuno-precipitation by HA-Trap_M beads. Total proteins (input) and immuno-purified proteins (IP) were separated on an SDS-PAGE gel followed by blotting. Anti-HA antibody was used to detect the immuno-precipitated HA fusion proteins and anti-Myc antibody was used to detect co-immuno-precipitated Myc-*Bn*SOBIR1 proteins, respectively.

Recently, the *A. thaliana* LRR-receptor-like kinase (LRR-RLKs). Suppressor of Bir1-1 (*At*Sobir1) and its homologs from tomato were shown to interact specifically with a number of LRR-receptor like proteins (LRR-RLPs), such as tomato Cf-4 and Ve1, and play a role in LRR-RLP-mediated resistance against the corresponding fungal pathogen (Liebrand et al., 2013, 2014). We searched the genome sequence of the *B. napus* cultivar ‘Darmor-*bzh*’ (Chalhoub et al., 2014) using the AtSOBIR1 as a query and six predicted *B. napus* SOBIR1 homologs (BnSOBIR1s) were identified. Based on phylogenetic analysis of the six BnSOBIR1s, as well as AtSBOIR1 and NbSOBIR1 (**Supplementary Figure S1**), two of the *B. napus* orthologs of SOBIR1, BnSOBIR1-A3 (BnaA03g1476) and BnSOBIR1-C3 (BnaC03g17800D), had the closest homology to AtSOBIR1. *BnSOBIR1-A3* and *BnSOBIR1-C3* were subsequently cloned to examine their interaction with LepR3. BnSOBIR1 proteins fused at the C-terminus to the Myc epitope tag and LepR3 fused at the C-terminus to the HA epitope tag were generated and transiently co-expressed in *N. benthamiana* leaves to perform co-immunopurification experiments. Myc-tagged BnSobir1-A3 and BnSobir1-C3 were co-immunopurified with LepR3 when HA-tagged LepR3 was used to capture the SOBIR1 complex (**Figure 4**).

### NbSOBIR1 and NbSERK3 (BAK1) are Required for AvrLm1/LepR3-Induced Cell Death Response in *N. benthamiana*

**FIGURE 5:**
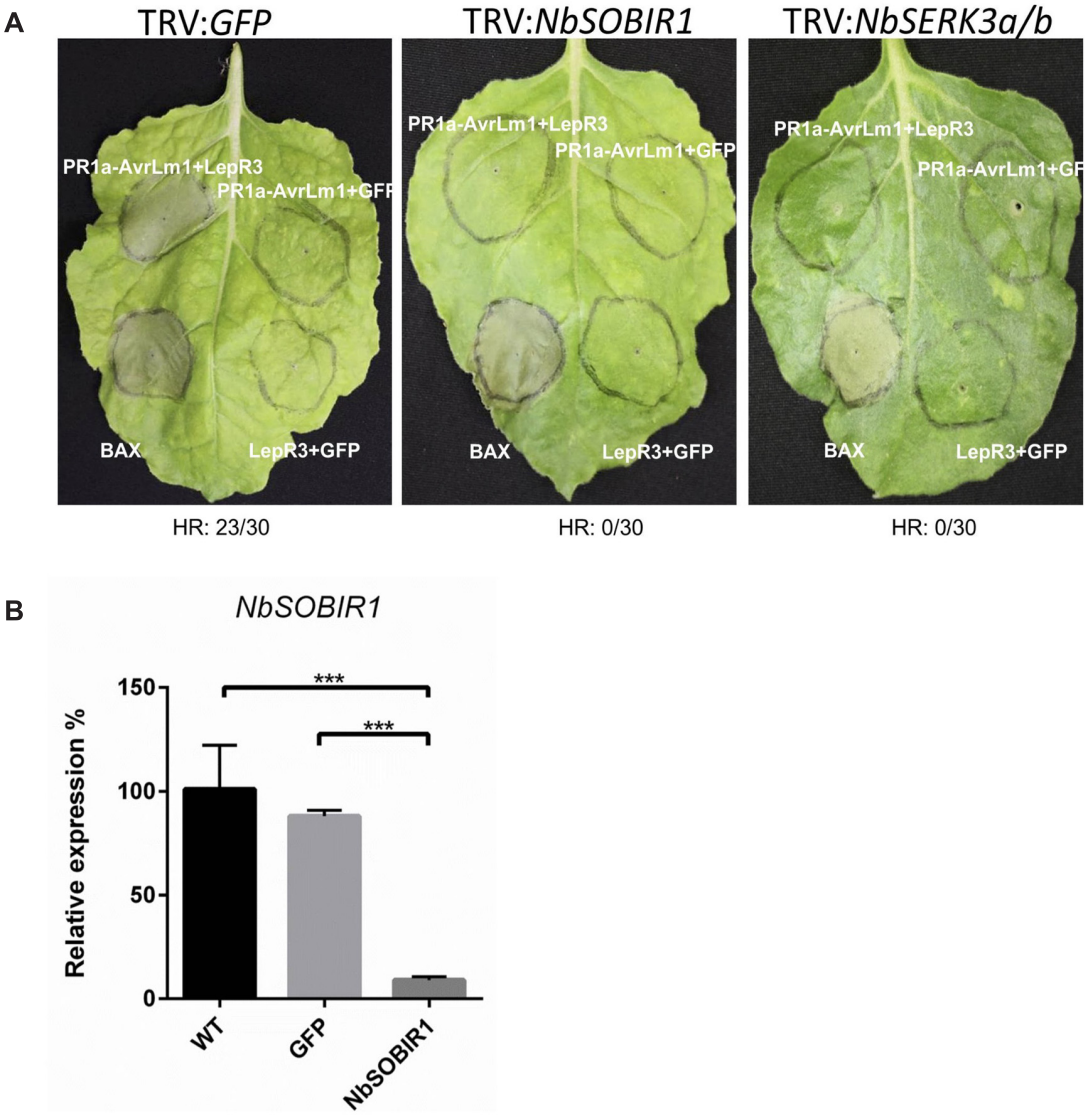
**NbSOBIR1 and NbSERK3 (BAK1) are required for LepR3-mediated development of cell death. (A)** 2-week-old *N. benthamiana* seedlings were subjected to virus-induced gene silencing (VIGS) by inoculation with the TRV:*NbSOBIR1*, TRV: *NbSERK3(BAK1)*, and TRV:*GFP*, respectively. TRV:*GFP* served as a control. Three weeks after TRV inoculation, *PR1a-AvrLm1/LepR3, PR1a-AvrLm1*/*GFP*, and *LepR3*/*GFP* were transiently co-expressed in the order indicated on the image. Cell death caused by Transiently expressed *BAX* served as a control. Leaves were photographed 6 days after infiltration. The experiment was repeated three times with 10 plants for each TRV construct. Numbers below the panels indicate the occurrence or the absence of HR within 30 sites co-infiltrated with *PR1a-AvrLm1/LepR3*. **(B)** qRT-PCR analysis showing *NbSOBIR1* expression in *N. benthamiana* inoculated with the indicated *TRV* VIGS constructs (TRV:*GFP* and TRV:*NbSOBIR1*) and in wildtype (WT) *N. benthamiana* without TRV inoculation. *NbSOBIR1* gene expression levels are normalized to that of actin. Values are means ± SE of triplicate reactions of three independent biological samples. Significant differences are represented by three asterisks (*P* < 0.001). Statistical differences (*P* <= 0.001) between groups were calculated by a one-way ANOVA and different groupings are indicated. Error bars represent the standard deviation.

The observation that the two SOBIR1 homologs from *B. napus* interact with LepR3 (**Figure 4**) suggests that both proteins play a role in *LepR3*-mediated defense signaling in plants. It has been reported that the AtSOBIR1 homolog from *N. benthamiana* (NbSOBIR1) was required for Avr4/Cf4 and Ave1/Ve1-triggered HR in tobacco plants (Liebrand et al., 2013; Fradin et al., 2014). We hypothesized that the NbSOBIR1, which exhibits closest homology with BnSOBIR1-A3 and -C3 (**Supplementary Figure S1**), is also required for AvrLm1/LepR3-triggered HR in *N. benthamiana*. To test this hypothesis, a recombinant TRV-based construct containing *NbSOBIR1* was used to knockdown the expression of *NbSOBIR1* homologs by VIGS. The same construct for *NbSOBIR1* as reported by Liebrand et al. (2013) was used and the function of this silencing construct to knockdown the expression of *NbSOBIR1* gene homologs has been shown to block Cf4-Avr4-mediated HR in *N. benthamiana* (Liebrand et al., 2013). A silencing construct targeting the tobacco *phytoene desaturase* (*PDS*) was included to monitor VIGS silencing efficiency and progression (VIGS of PDS causes photo-bleaching). A TRV:*GFP* construct was used as a negative control. Three weeks after viral inoculations, photobleaching symptoms were observed in all of the *PDS* silenced *N. benthamiana* plants confirming the onset of VIGS (**Supplementary Figure S2**). The *N. benthamiana NbSOBIR1*-silenced (TRV:*NbSOBIR1*) plants were infiltrated to transiently co-express *PR1a-AvrLm1/LepR3; PR1a-AvrLm1/GFP; and LepR3/GFP*. In TRV:*NbSOBIR1* plants, *PR1a*-*AvrLm1/LepR3*-triggered HR was severely compromised (**Figure 5A**). However, in the control plant (TRV:*GFP*), HR induced by the co-expression *PR1a*-*AvrLm1/LepR3* was not affected (**Figure 5A**). In addition, qRT-PCR confirmed that *NbSOBIR1* expression levels were ∼90% reduced upon inoculation with TRV:*NbSOBIR1* compared with the one inoculated with TRV:*GFP* or the wildtype (WT) *N. benthamiana* without TRV inoculation (**Figure 5B**). Furthermore, to verify TRV:*NbSOBIR1* plants were not compromised in their ability to mount programmed cell death, the proapoptotic factor Bcl2-Associated protein X (BAX) was transiently expressed in TRV:*NbSOBIR1* plants (Lacomme and Santa Cruz, 1999). Expression of BAX triggered a strong cell death in SOBIR1-silenced plants (**Figure 5A**), confirming that the ability of these plants to mount programmed cell death was not compromised.

Recently, it has been reported that BRI1-ASSOCIATED KINASE 1 (BAK1)/SOMATIC EMBRYOGENESIS RECEPTOR KINASE (SERK)3 associates with Cf-4 and Cf-9 upon elicitation with the matching effector ligands Avr4 and Avr9 (Postma et al., 2015). In addition, BAK1-silenced *N. benthamiana* plants were compromised in Avr4-triggered endocytosis and effector-triggered cell death. BAK1 is also required for resistance of Cf-4 tomato plants against *C. fulvum* carrying *Avr4* (Postma et al., 2015). To determine if the components of Cf4 receptor complex are also common to the LepR3 receptor complex, the requirement for *BAK1/SERK3* in AvrLm1/LepR3-triggered cell death in *N. benthamiana* plants was investigated. The AvrLm1/LepR3-triggered cell death was examined by co-expression of these constructs in *N. benthamiana* NbSERK3-silenced plants. The effectiveness of the silencing construct *TRV2: SERK3a/b (BAK1)* to knockdown the expression of *NbSERK3a/b* homologs in *N. benthamiana* has been shown (Chaparro-Garcia et al., 2011; Postma et al., 2015). The TRV:*GFP* construct was used as a negative control. Three weeks after viral inoculation, the tobacco *NbSERK3a/b*-silenced (TRV:*NbSERK3*) plants were infiltrated to transiently co-express *PR1a*-*AvrLm1/LepR3, PR1a-AvrLm1/GFP, and LepR3/GFP*. The PR1a-AvrLm1/LepR3-triggered cell death was compromised in *NbSERK3a/b*-silenced plants, as compared to *TRV:GFP* control plants (**Figure 5A**).

## Discussion

Race-specific resistance against *L. maculans* remains the only practical approach to control blackleg disease of canola (Raman et al., 2013). The genetics of this race-specific resistance have been studied in detail; however, the molecular mechanism of *L. maculans* perception by *B. napus* remained unknown until the cloning of *LepR3* and *Rlm2*, two *B. napus R* genes against blackleg (Larkan et al., 2013; Larkan et al., 2015). Here we presented further studies to unravel the components of LepR3-AvrLm1 recognition complex. Since most of the tools developed for the functional analysis of *R-Avr* genes cannot be applied to *B. napus*, transient expression in *N. benthamiana* was used to study AvrLm1-LepR3 interaction. Using this model plant, two LRR-RLK proteins, SOBIR1 and BAK1 were identified as components of the LepR3 recognition complex. The requirement of SOBIR1 for plant immunity initiated by RLP proteins has been shown for the tomato Cf4, Cf9, and Ve1 which are effective against *C. fulvum* and *V. dahlia*, respectively (Liebrand et al., 2014). The kinase domain of SOBIR1 is suggested to be involved in downstream signaling. Indeed, both the kinase and LRR domains of SOBIR1 are required for Cf4/Avr4-induced HR, although they are dispensable for interaction with Cf4 (Bi et al., 2015). In addition several RLPs from *A. thaliana* form a complex with the AtSOBIR1. SOBIR1 bind to the AtRLP23, an *Arabidopsis* LRR-RLP that perceives the Necrosis and ethylene-inducing peptide 1-like proteins (NLPs), a conserved protein in many prokaryotic and eukaryotic microorganisms (Albert et al., 2015). AtRLP42 that was identified as RESPONSIVENESS TO BOTRYTIS POLYGALACTURONASES1 (RBPG1) was shown to bind AtSOBIR1 (Zhang et al., 2014). Another *Arabidopsis* immune receptor is RLP30 that perceives the SCLEROTINIA CULTURE FILTRATE ELICITOR1 (SCFE1) from *Sclerotinia sclerotiorum* and is dependent on SOBIR1 for its function (Zhang et al., 2013). Six paralogs of SOBIR1 (BnSOBIR1s) are present in *B. napus* and LepR3 interacted with two BnSOBIR1 which had closest similarity to AtSOBIR1. This findings indicates that a degree of functional redundancy exists and also that other BnSOBIR1 are likely part of receptor complexes for the presently unknown RLP against *L. maculans*. The interaction of LepR3 with SOBIR1 in the absence of AvrLm1 suggests that this interaction is not ligand-dependent, as has also been reported for the interaction of Cf proteins with SOBIR1 (Postma et al., 2015). It has been documented that NbSOBIR1 is required for Cf4- or Ve1-mediated cell death in *N. benthamiana* plants (Liebrand et al., 2013). These findings show the presence of conserved mechanisms for the perception of apoplastic effectors in two distinct host-pathogen systems.

BAK1 was demonstrated to be another component of the LepR3-SOBIR1 recognition complex. The requirement of BAK1 for HR initiated by LepR3/AvrLm1 supports similar findings reported for the recognition of *C. fulvum* apoplastic effectors Avr4/9 by the tomato RLPs Cf4/9 (Postma et al., 2015). We recently reported that SOBIR1 interacts with the second *B. napus* R protein Rlm2 that recognizes *L. maculans* AvrLm2 effector proteins (Ghanbarnia et al., 2015; Larkan et al., 2015). It is likely that BAK1 is also required for recognition of AvrLm2 by Rlm2. BAK1 is best known as being required for the perception of the bacterial PAMP, flg22, through interaction with the FLS2 receptor complex (Chinchilla et al., 2007). However, data that we have presented here and that by Postma et al. (2015) clearly shows the importance of BAK1 in ETI against the apoplastic fungi and highlights the common mechanisms involved in the recognition of PAMPs and the recognition of effectors from extracellular fungi. Accumulating evidence suggests that common features shared between PTI and ETI blur the distinction between these two immunity systems (Thomma et al., 2011). Several PAMPs and effectors are discussed in a recent review by Cook et al. as examples that deviate from the currently accepted definition of PTI/ETI (Cook et al., 2015). A more relevant example to our study is the case of Cf4/Avr4. It was suggested that Cf4 is a PRR that recognizes conserved chitin binding domains present in *C. fulvum* Avr4 and its homolog in *Mycosphaerella fijiensis*, a pathogen that causes the black leaf streak disease in banana (Stergiopoulos et al., 2010). However, AvrLm1, and AvrLm2 both lack any known functional or conserved domains, a feature common to most effector proteins. In addition, AvrLm1 and AvrLm2 have other features typical of fungal and oomycete effectors, such as being located in the AT-rich block of the genome, and showing allelic variation caused by point mutation (*AvrLm2*) or complete deletion (*AvrLm1*; Gout et al., 2006; Ghanbarnia et al., 2015).

We also exploited transient expression in *N. benthamina* to identify the effector domain of AvrLm1 and the results validated by expressing the various truncated *AvrLm1* constructs in *L. maculans* and conducting pathology assays on the host plant *B. napus*. Based on these analyses, only the first 18 amino acids of the AvrLm1 mature protein is dispensable for its function to induce LepR3-dependent HR. Deletion in the C-terminal part of AvrLm1 disrupts the LepR3 mediated HR. The C-terminal region of effectors from plant pathogenic oomycetes and fungi has been shown to act as effector domain. The *AvrLm1* gene is located in a large AT-rich, heterochromatin-like region that is mostly devoid of other *L. maculans* coding sequences (Rouxel et al., 2011). Furthermore, the genome environment surrounding *AvrLm1* is enriched with transposons elements (TEs), which is thought to contribute to allele diversification. The *AvrLm1* allele is entirely deleted in *L. maculans* isolates virulent on *B. napus* cultivars containing *LepR3* or *Rlm1* (Gout et al., 2006; Rouxel et al., 2011). AvrLm1 contains only one cysteine residue, which is unusual for effectors secreted to the apoplast of host plants. However, we demonstrated by transient expression in *N. benthamiana* and transformation of *L. maculans* that AvrLm1 must be secreted to the host apoplast to be perceived by LepR3. Still, it is possible that AvrLm1 is translocated at some stage into the plant host cells to modulate host immunity. Another *B. napus R* gene (*Rlm1*) encoding Rlm1 that also recognizes *AvrLm1* product but is physically distinct from *LepR3*. We have not been able to detect the direct interaction between AvrLm1 and LepR3 (unpublished data) pointing to the possibility that AvrLm1 interacts with a presently unknown host protein that is commonly guarded by Rlm1 and LepR3. Determining the structure of AvrLm1 could help to determine surface residues required for the interaction with host plant targets.

The information presented here underlines the importance of understanding the molecular function of *B. napus* receptor complexes against *L. maculans* in developing durable resistance against this rapidly evolving pathogen. We demonstrated for the first time that *N. benthamiana* model system can be used to facilitate functional characterisation of *R/Avr* gene pairs from the *B. napus-L. maculans* pathogen system which is less amenable to genetic manipulation. These findings provide invaluable tools to analyze the function of additional *R* and *Avr* genes from the *B. napus-L. maculans* pathogen system that will be identified in future.

## Author Contributions

LM conducted the experiments. LM and MB designed the experiments, analyzed the data and wrote the manuscript. Both authors provided intellectual input, approved the manuscript and are accountable for accuracy and integrity of this study.

## Conflict of Interest Statement

The authors declare that the research was conducted in the absence of any commercial or financial relationships that could be construed as a potential conflict of interest.

## Supplementary Material

Figure S1 **Virus-induced gene silencing (VIGS) of *phytoene desaturase* (*PDS*) gene in *Nicotiana benthamiana.* VIGS of the *phytoene desaturase* (*PDS*) gene leads to photobleaching in leaves of *N. benthamiana* plant. Pictures were taken at 3 weeks after inoculation**.

Figure S2 **Phylogenetic tree of SOBIR1 from *Arabidopsis, Brassica napus* and *N. benthamiana*. The protein sequences of AtSBOIR1, BnSOBIR1-A3, -C3,-A4a,-C4a,-A4b,-C4b, and NbSOBIR1 were aligned and analyzed with CLC for the phylogenetic tree**.
