# Supplementary material for: The receptor-like kinase SOBIR1 interacts with Brassica napus LepR3 and is required for Leptosphaeria maculans AvrLm1-triggered immunity
Source: Front Plant Sci. 2015 Oct 29;6:933. doi: 10.3389/fpls.2015.00933 (PMC4625043; doi:10.3389/fpls.2015.00933)
Supplement: Supplementary file 1 [file Table_1.DOCX]

Supplementary table 1

Primers used in this study

| **Primer names** | **Sequences** |
| --- | --- |
| AvrLm1-Up-F | CGCGGTACCCACCTCTGTACTAAGGTTACT |
| AvrLm1-Up-R | CACGGTACCGTTGGTAATTGTGTTTAACAA |
| TubA-F | CGCTTAATTAA AGTAGATGCCGACCGGGATCC |
| TubA-R | CACTTAATTAAGACGGCCAGTGCCAAGCTTGA |
| LepR3-FB | ACAAGTTTGTACAAAAAAGCAGGCTTCATGAAAGGCTCTGTGAAATCATTTAGTCTC |
| LepR3-RB-s | ACCACTTTGTACAAGAAAGCTGGGTCACGACGTGTGGTGCTTATTCTTCTG |
| AvrLm1-FB | ACAAGTTTGTACAAAAAAGCAGGCTTTATGGTTCAATTCAAGACTATCTTTCTATCA |
| AvrLm1-RB | ACCACTTTGTACAAGAAAGCTGGGTCTTATATTGCACCCGCAATATCAAATTTT |
| dspAvrLm1-FB | ACAAGTTTGTACAAAAAAGCAGGCTTTATGTCCCCAGCTACCAAGAACAATGTGA |
| BnSORB1-A3C3-FB | ACAAGTTTGTACAAAAAAGCAGGCTTCATGGCTGTTCCCACAGGTCTCTTTC |
| BnSORB1-A3C3-RB-S | ACCACTTTGTACAAGAAAGCTGGGTCGTGTTTGATCTGGGACAGCATAGTC |
