# Supplementary figures and images for: The receptor-like kinase SOBIR1 interacts with Brassica napus LepR3 and is required for Leptosphaeria maculans AvrLm1-triggered immunity
Source: Front Plant Sci. 2015 Oct 29;6:933. doi: 10.3389/fpls.2015.00933 (PMC4625043; doi:10.3389/fpls.2015.00933)

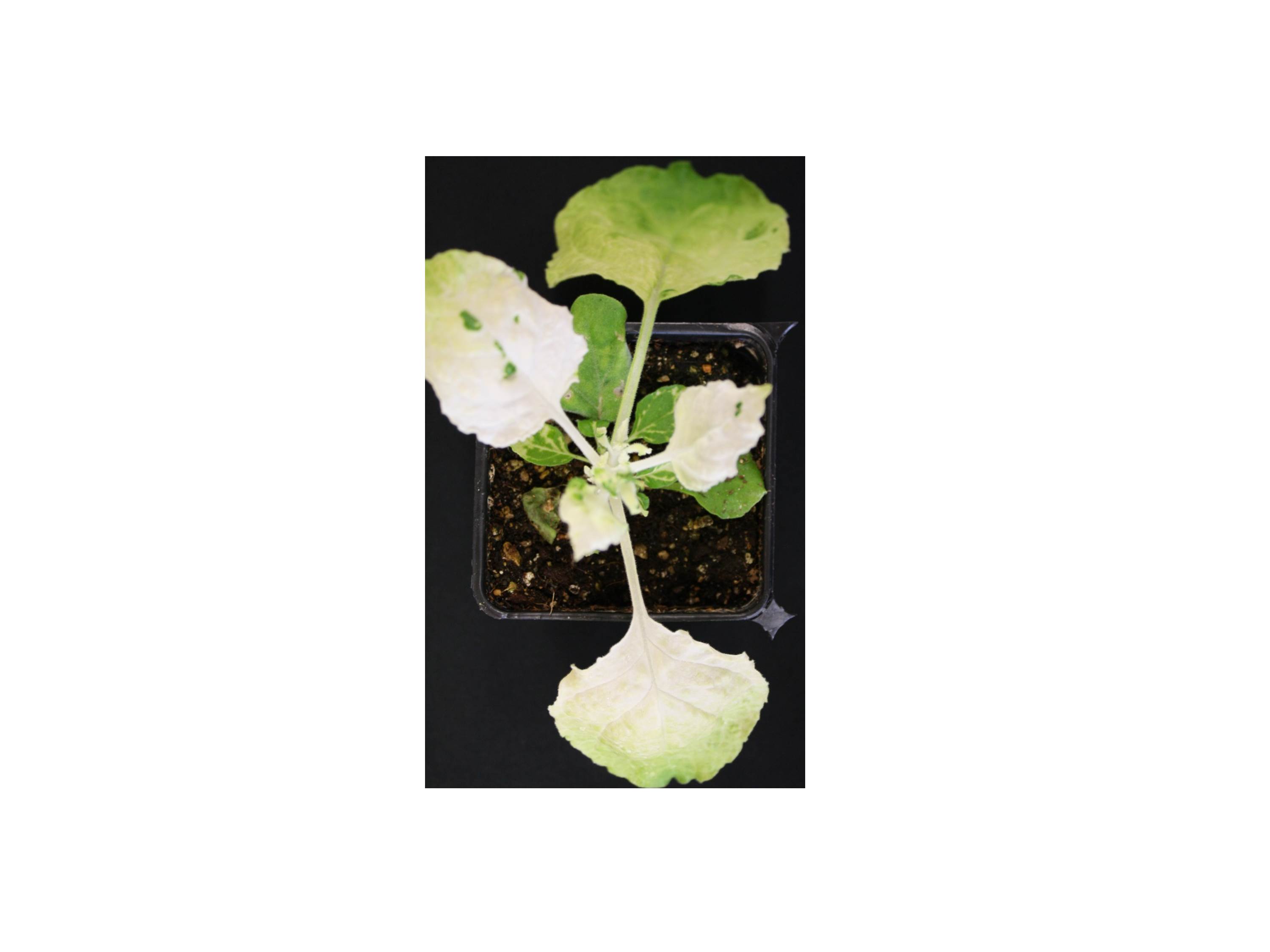

Supplement: Figure S1 — Virus-induced gene silencing (VIGS) of phytoene desaturase (PDS) gene in Nicotiana benthamiana. VIGS of the phytoene desaturase (PDS) gene leads to photobleaching in leaves of N. benthamiana plant. Pictures were taken at 3 weeks after inoculation. [file Image_1.JPEG]

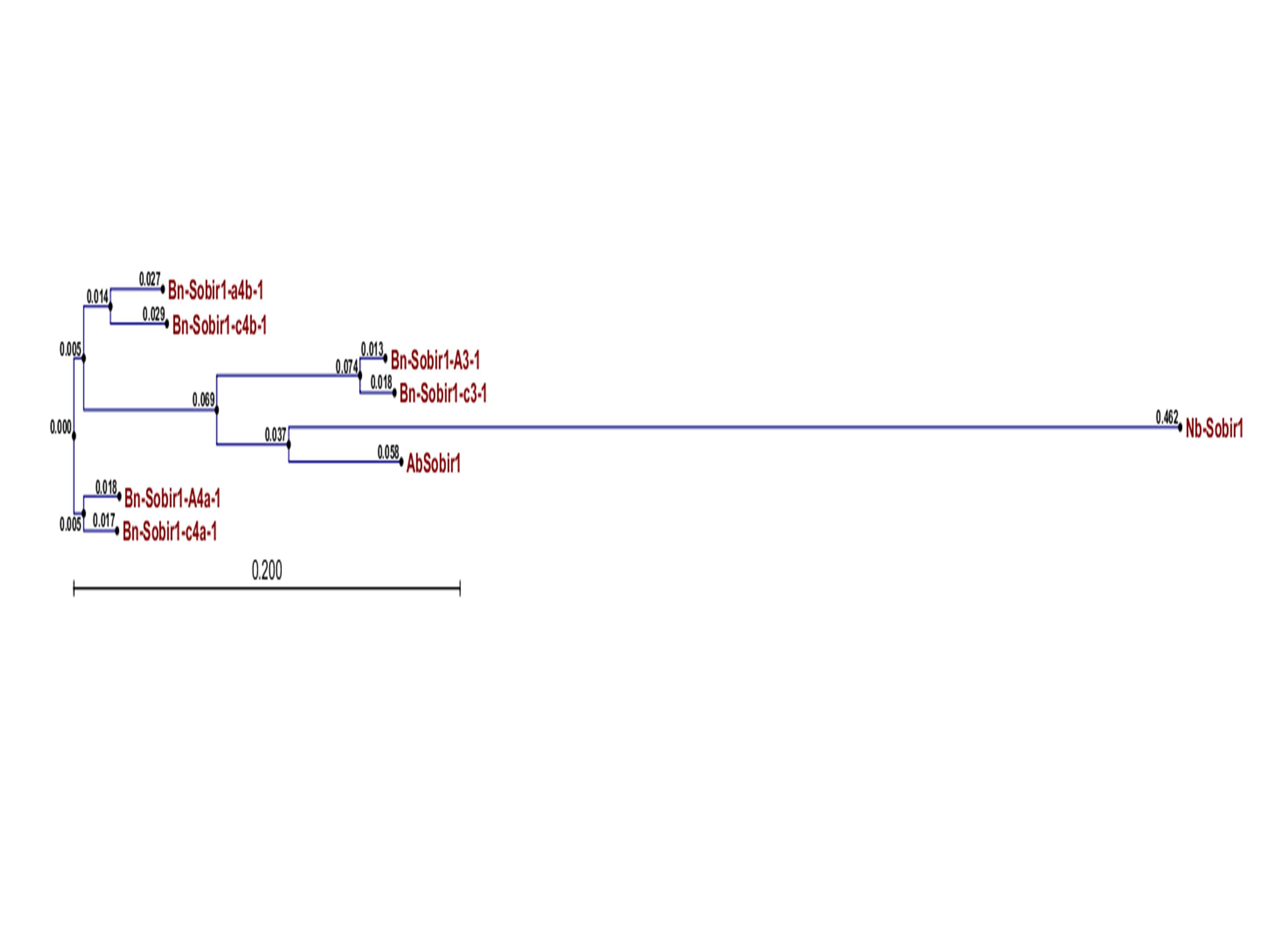

Supplement: Figure S2 — Phylogenetic tree of SOBIR1 from Arabidopsis, Brassica napus and N. benthamiana. The protein sequences of AtSBOIR1, BnSOBIR1-A3, -C3,-A4a,-C4a,-A4b,-C4b, and NbSOBIR1 were aligned and analyzed with CLC for the phylogenetic tree. [file Image_2.JPEG]
